# A molecular phylogeny of *Dorylus *army ants provides evidence for multiple evolutionary transitions in foraging niche

**DOI:** 10.1186/1471-2148-7-56

**Published:** 2007-04-04

**Authors:** Daniel JC Kronauer, Caspar Schöning, Lars B Vilhelmsen, Jacobus J Boomsma

**Affiliations:** 1 Institute of Biology, Department of Population Biology, University of Copenhagen, Universitetsparken 15, 2100 Copenhagen, Denmark; 2 Zoological Museum, University of Copenhagen, Universitetsparken 15, 2100 Copenhagen, Denmark

## Abstract

**Background:**

Army ants are the prime arthropod predators in tropical forests, with huge colonies and an evolutionary derived nomadic life style. Five of the six recognized subgenera of Old World *Dorylus *army ants forage in the soil, whereas some species of the sixth subgenus (*Anomma*) forage in the leaf-litter and some as conspicuous swarm raiders on the forest floor and in the lower vegetation (the infamous driver ants). Here we use a combination of nuclear and mitochondrial DNA sequences to reconstruct the phylogeny of the *Dorylus s.l*. army ants and to infer the evolutionary transitions in foraging niche and associated morphological adaptations.

**Results:**

Underground foraging is basal and gave rise to leaf-litter foraging. Leaf-litter foraging in turn gave rise to two derived conditions: true surface foraging (the driver ants) and a reversal to subterranean foraging (a clade with most of the extant *Dorylus s.s*. species). This means that neither the subgenus *Anomma *nor *Dorylus s.s*. is monophyletic, and that one of the *Dorylus s.s*. lineages adopted subterranean foraging secondarily. We show that this latter group evolved a series of morphological adaptations to underground foraging that are remarkably convergent to the basal state.

**Conclusion:**

The evolutionary transitions in foraging niche were more complex than previously thought, but our comparative analysis of worker morphology lends strong support to the contention that particular foraging niches have selected for very specific worker morphologies. The surprising reversal to underground foraging is therefore a striking example of convergent morphological evolution.

## Background

Army ants are functionally defined by a suite of interrelated behavioural and morphological traits [1]: they are obligate group predators, they frequently emigrate to new nest sites, and their queens are permanently wingless and highly specialized egg-layers. Some species conduct spectacular swarm raids, in which hundreds of thousands of ants form a dense carpet that sweeps through areas of 1000 m^2 ^or more on a single day in search of prey. However, this remarkable foraging behaviour is found in rather few species, as most army ants have an inconspicuous, completely underground lifestyle.

Army ants in the dorylomorph clade (sometimes referred to as the "true" army ants) form a monophyletic group that originated in Gondwana in the mid-Cretaceous, slightly over 100 million years ago [2,3]. With the subsequent break-up of this supercontinent, the clade split into the New World army ants (subfamily Ecitoninae) and the Old World army ants. The latter comprises three monophyletic subfamilies, the Dorylinae (with a primarily Afrotropical distribution), the Aenictinae (primarily distributed in Asia and the Oriental region with fewer African species), and the enigmatic subfamily Aenictogitoninae, which is only known from nocturnal males that have been sporadically collected at lights in sub-Saharan Africa, and which has been inferred to be the sister taxon to the Dorylinae [2,4,5]. Whether the Aenictogitoninae have all the defining army ant traits will thus not be known until colonies with workers and queens have been discovered.

The subfamily Dorylinae consists of the single genus *Dorylus*, which has been subdivided into six subgenera [6,7]. This subdivision has, however, not become generally accepted [8]. Five of these subgenera are entirely subterranean, but the sixth subgenus *Anomma *encompasses both species that hunt in the leaf-litter and species that forage on the forest floor and in the vegetation. The latter are the infamous "driver ants" [9], renown for their massive swarm raids that overwhelm most invertebrates that do not get out of their way and occasionally also small vertebrates [1].

A major reason for the confused taxonomy and classification of *Dorylus s.l*. is that most of the recognized species have only been described from a single caste or sex [7]. The large and distinctive males, which were originally described by Linné [10] as *Vespa *wasps and are known in Africa as "sausage flies", are easily collected at light sources during the night, but have rarely been taken from nests together with workers or queens. The extreme worker polymorphism within colonies and the difficulty of finding the largest workers among the foragers of subterranean species poses an additional challenge to resolving the phylogenetic relationships between species and species-groups with morphological data, because valid comparisons of homologous character states can only be made between workers of the same size. Finally, similar worker morphologies in different species might reflect convergent adaptations to specific foraging niches and prey spectra rather than common ancestry.

In the present study we use DNA sequence data to overcome the problems mentioned in the previous paragraph and to obtain an accurate and unbiased phylogeny for the genus *Dorylus*. We use this phylogeny to reconstruct the evolution of the driver ants from their subterranean ancestors, and to infer the morphological changes that have occurred as putative adaptations to changes in foraging niche. The DNA sequences also allow us to establish new associations between males and workers for several species across the different subgenera, thus helping to clarify *Dorylus *taxonomy.

## Results

### Phylogenetic analyses

**Figure 1:**
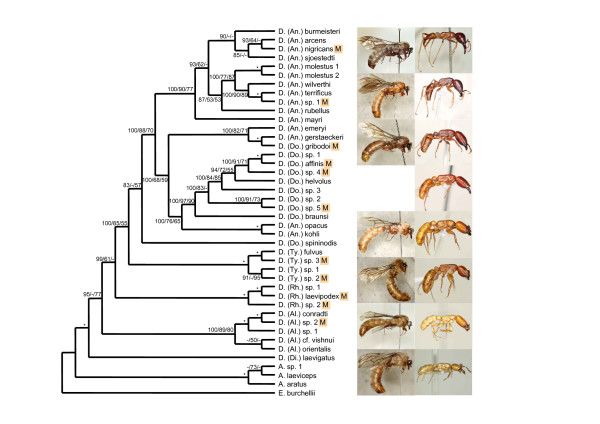
**Maximum likelihood topology from the analysis of the combined data for all taxa**. Subgenera abbreviations: *Anomma *(An.), *Dorylus s.s*. (Do.), *Typhlopone *(Ty.), *Rhogmus *(Rh.), *Alaopone *(Al.), *Dichthadia *(Di.). Male specimens are marked with an "M" next to the specimen label. The numbers on each branch are Bayesian posterior probabilities/ML bootstraps/MP bootstraps. Nodes that received a support of 100 in every analysis are indicated by an asterisk, whereas support values lower than 50 are indicated by a hyphen. Pictures to the right depict males and workers of representative species for all major groups (not on the same scale); from top to bottom: 1. *D.(An.) terrificus*, 2. *D. (Do.) gribodoi *and *D. (An.) gerstaeckeri *(possibly the same species), 3. *D. (Do.) affinis*, 4. *D. (Do.) spininodis *(male unknown), 5. *D. (Ty.) fulvus*, 6. *D. (Rh.) laevipodex*, 7. *D. (Al.) conradti*, 8. *D. (Di.) laevigatus*).

The maximum likelihood (ML) topology (likelihood score 17554.6) is given in Fig. 1 together with Bayesian posterior probabilities and ML and maximum parsimony (MP) bootstrap values for all nodes. All three methods recovered very similar topologies: disagreements were restricted to a few terminal taxa, mainly within the driver ant clade, and none of these affected the conclusions to be drawn.

The total evidence indicates that: 1. The Southeast Asian monotypic subgenus *Dichthadia *is sister to the remaining *Dorylus s.l*. species (although the respective node was collapsed in ML bootstrap analysis), confirming an earlier inference by Brady [2]. 2. Each of the subgenera *Alaopone*, *Typhlopone*, and *Rhogmus *is monophyletic according to the taxon sample obtained for the present study. 3. The species of the subgenus *Anomma *that forage in the leaf-litter are more closely related to subterranean species of the subgenus *Dorylus s.s*., than to the surface swarm foraging *Anomma *driver ants, which form a well supported clade. The close phylogenetic relationship between the leaf-litter *Anomma *species and the subgenus *Dorylus s.s*. (without *D. spininodis *– see below) also emerges from the position of the male of *D. gribodoi*, which clearly belongs to a clade of leaf-litter *Anomma *species, although it was originally described as belonging to the subgenus *Dorylus s.s*.. 4. While most species of the subgenus *Dorylus s.s*. form a clade nested within the leaf-litter *Anomma *species, *D. (Dorylus) spininodis *is more distantly related and may be the sister taxon to the remaining species of *Dorylus s.s*. plus *Anomma *(although this inferred relationship has low statistical support and *D. spininodis *may even be more distantly related; Fig. 1). This implies that the subgenus *Anomma *is paraphyletic and the subgenus *Dorylus s.s*. is polyphyletic.

### Male-worker associations

Pair-wise genetic distances of mitochondrial sequences between closely related male-worker pairs were clearly bimodally distributed with five values ≤ 0.003 and the remaining five values ≥ 0.044 (Table 1). Using a 0.014 reference value for two distantly related specimens of *Dorylus (Anomma) molestus *from the same population, we concluded that the first five pairs represent workers and males of the same species. Some of the latter pairs may eventually turn out to belong to the same species as well once additional data become available, because most of these worker and male specimens came from distant populations (Table 2) so that our threshold value for sympatric conspecifics is probably very conservative.

**Table 1:** Uncorrected pair-wise genetic distances between closely related Dorylus s.l. workers and males.

Worker	Male	p-Distance
**D. (An.) molestus 1 and 2**	**-**	**0.014**
D. (Rh.) sp. 1	D. (Rh.) laevipodex	0.000*
D. (An.) terrificus	D. (An.) sp. 1	0.000*
D. (Al.) conradti	D. (Al.) sp. 2	0.002*
D. (Ty.) fulvus	D. (Ty.) sp. 3	0.002*
D. (Do.) sp. 1	D. (Do.) affinis	0.003*
D. (Ty.) sp. 1	D. (Ty.) sp. 2	0.044
D. (An.) arcens	D. (An.) nigricans	0.048
D. (An.) burmeisteri	D. (An.) nigricans	0.055
D. (An.) gerstaeckeri	D. (Do.) gribodoi	0.063
D. (Do.) sp. 2	D. (Do.) sp. 5	0.073

Genetic distances were calculated from the mitochondrial DNA sequences. The distance between two worker specimens of *Dorylus (Anomma) molestus *from the same population at Mt. Kenya is included as a reference (in bold). Pair-wise distances below 0.014 were inferred to characterize pairs that belong to the same species and are marked with an asterisk.

**Table 2:** List of specimens used for DNA sequencing.

Species/label	Sub-genus	Foraging niche	Specimen	Collection locality	GenBank accession numbers				Museum voucher no
						
					*COI1*	*COI2*	*COII*	*wingless*	
wilerthi	An	surface swarms	worker	Kibale (Uganda)	EF413837	EF413761	EF413798	EF413874	1
terrificus	An.	surface swarms	worker	Kibale (Uganda)	EF413842	EF413767	EF413804	EF413880	2
molestus 1	An.	surface swarms	worker	Mt. Kenya (Kenya)	EF413809	EF413732	EF413772	EF413848	3
molestus 2	An.	surface swarms	worker	Mt. Kenya (Kenya)	EF413836	EF413760	EF413797	EF413873	4
burmeisteri	An.	surface swarms	worker	Comoé (Ivory Coast)	EF413808	EF413731	EF413771	EF413847	5
mayri	An.	surface swarms	worker	Bossou (Guinea)	EF413844	-	-	EF413882	6
arcens	An.	surface swarms	worker	Taï (Ivory Coast)	EF413829	EF413753	EF413791	EF413867	7
sjoestedti	An.	surface swarms	worker	Ndoki (DR Congo)	EF413834	EF413758	EF413795	EF413872	8
rubellus	An.	surface swarms	worker	Gashaka (Nigeria)	EF413833	EF413757	-	EF413871	9
emeryi	An.	leaf-litter	worker	Taï (Ivory Coast)	EF413810	EF413733	EF413773	EF413849	10
gerstaeckeri	An.	leaf-litter	worker	Bossou (Guinea)	EF413812	EF413736	EF413776	EF413852	11
opacus	An.	leaf-litter	worker	Kibale (Uganda)	EF413813	EF413737	EF413777	EF413853	12
kohli	An.	leaf-litter	worker	Kibale (Uganda)	EF413814	EF413738	EF413778	EF413854	13
nigricans	An.	n. a.	male	Taï (Ivory Coast)	EF413841	EF413766	EF413803	EF413879	14
sp. 1	An.	n. a.	male	Kibale (Uganda)	EF413843	EF413768	EF413805	EF413881	15
sp. 1	Do.	subterranean	worker	Mt. Kenya (Kenya)	EF413811	EF413735	EF413775	EF413851	16
sp. 2	Do.	subterranean	worker	Bossou (Guinea)	EF413815	EF413739	EF413779	EF413855	17
helvolus	Do.	subterranean	worker	Cullinan (South Africa)	EF413832	EF413756	EF413794	EF413870	18
braunsi	Do.	subterranean	worker	Kakamega (Kenya)	EF413835	EF413759	EF413796	-	19
sp. 3	Do.	subterranean	worker	Gashaka (Nigeria)	EF413827	EF413751	-	EF413865	20
spininodis	Do.	subterranean	worker	Gashaka (Nigeria)	EF413826	EF413750	EF413789	EF413864	21
gribodoi	Do.	n. a.	male	Taï (Ivory Coast)	EF413817	EF413741	EF413781	EF413857	22
affinis	Do.	n. a.	male	Mt. Kenya (Kenya)	EF413846	EF413770	EF413807	EF413884	23
sp. 4	Do.	n. a.	male	Mt. Kenya (Kenya)	EF413816	EF413740	EF413780	EF413856	24
sp. 5	Do.	n. a.	male	Gashaka (Nigeria)	EF413845	EF413769	EF413806	EF413883	25
sp. 1	Rh.	subterranean	worker	Mt. Kenya (Kenya)	EF413818	EF413742	EF413782	EF413858	26
laevipodex	Rh.	n. a.	male	Mt. Kenya (Kenya)	EF413840	EF413764	EF413801	EF413877	27
sp. 2	Rh.	n. a.	male	Gashaka (Nigeria)	EF413831	EF413755	EF413793	EF413869	28
laevigatus	Di.	subterranean	worker	Poring (Malaysia)	EF413819	EF413743	EF413783	AY233632	29
fulvus	Ty.	subterranean	worker	Mt. Kenya (Kenya)	-	EF413734	EF413774	EF413850	30
sp. 1	Ty.	subterranean	worker	Gashaka (Nigeria)	EF413828	EF413752	EF413790	EF413866	31
sp. 2	Ty.	n. a.	male	Ile-Ife (Nigeria)	EF413838	EF413762	EF413799	EF413875	32
sp. 3	Ty.	n. a.	male	Mt. Kenya (Kenya)	-	EF413765	EF413802	EF413878	33
conradti	Al.	subterranean	worker	Kakamega (Kenya)	EF413820	EF413744	-	EF413860	34
cf. vishnui	Al.	subterranean	worker	Poring (Malaysia)	EF413821	EF413745	EF413784	EF413861	35
orientalis	Al.	subterranean	?	?	AY233706	AY233706	-	AY233631	-
sp. 1	Al.	n. a.	male	Gashaka (Nigeria)	EF413830	EF413754	EF413792	EF413868	36
sp. 2	Al.	n. a.	male	Kakamega (Kenya)	EF413839	EF413763	EF413800	EF413876	37
Aenictus sp. 1	n. a.	n. a.	worker	Mt. Kenya (Kenya)	EF413822	EF413746	EF413785	EF413862	38
A. aratus	n. a.	n. a.	worker	Tawau Hills (Malaysia)	EF413824	EF413748	EF413787	AY233628	39
A. laeviceps	n. a.	n. a.	worker	Poring (Malaysia)	EF413823	EF413747	EF413786	AY233627	40
Eciton burchellii	n. a.	n. a.	worker	Henri Pittier (Venezuela)	EF413825	EF413749	EF413788	EF413863	41

Specimens have been deposited at the Zoological Museum in Copenhagen, numbered consecutively with a reference to this publication (Kronauer et al. 2007/1 – Kronauer et al. 2007/41). Abbreviations of subgenera are *Anomma *(An.), *Dorylus s.s*. (Do.), *Rhogmus *(Rh.), *Dichthadia *(Di.), *Typhlopone *(Ty.), and *Alaopone *(Al.). Foraging niches were not applicable (n. a.) for males and outgroup taxa.

### Reconstructing evolutionary transitions in foraging niche

**Figure 2:**
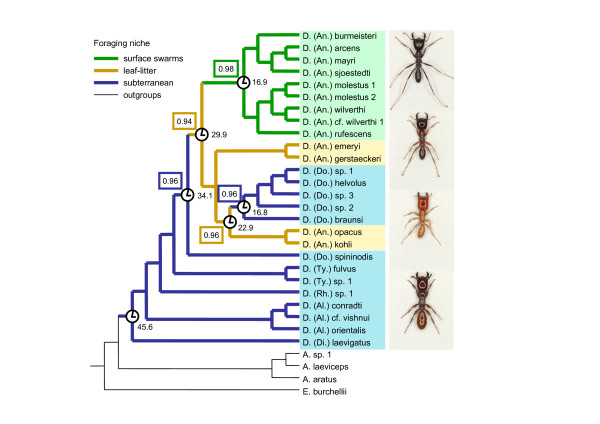
**The evolution of foraging niche in *Dorylus s.l*. army ants**. The most parsimonious reconstruction of the evolutionary transitions is illustrated by different branch colours, while the Bayesian reconstruction with the highest posterior probability is indicated with coloured rectangles at the relevant nodes. Age estimates (clocks) for key nodes are given in million years ago (mya), after fixing the most recent common ancestor of *Dorylus s.l*. at 45.6 mya [2]; see text for ranges. The phylogeny was obtained by Bayesian analysis of a dataset from which males had been excluded. Subgenera are abbreviated as in Fig. 1. Photographs to the right depict workers of representative species with different foraging niches and illustrate the differences in relative leg length, mandible length, and antennal scape length associated with the three foraging niches (all pictures are the same scale and all workers have a maximum head width of 2.27 mm; from top to bottom: 1. *D. (An.) arcens *(surface swarm raiding), 2. *D. (An.) gerstaeckeri *(leaf-litter), 3. *D. (Do.) affinis *(reversal subterranean) 4. *D. (Ty.) sp. 1 *(original subterranean).

MP and Bayesian analyses unambiguously reconstructed three transitions in foraging niche (Fig. 2). The first transition from subterranean to leaf-litter foraging took place between 34.1 and 29.9 million years ago (mya) (possible range: 47.9 – 19.8 mya) along the branch leading to the clade of *Anomma *plus *Dorylus s.s*. (without *D. spininodis*). The second transition, a reversal from leaf-litter foraging to subterranean foraging occurred between 22.9 and 16.8 mya (possible range: 32.2 – 11.3 mya) along the branch leading to the *Dorylus s.s*. clade (again without *D. spininodis*). Finally, a transition from leaf-litter foraging to surface swarm raiding occurred between 29.9 and 16.9 mya (possible range: 42.0 – 11.1 mya) along the branch leading to the driver ant clade.

### Morphological analyses

**Figure 3:**
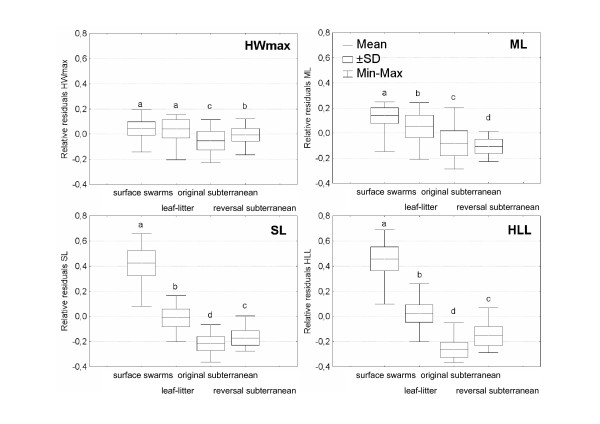
**Relative residuals from best-fit regression models over the common size distribution of workers for thirteen *Dorylus s.l*. species in the different foraging niche categories**. We distinguished between four categories of foraging niche: The surface swarm raiding *Anomma *driver ant species ("surface swarm"), the leaf-litter *Anomma *species ("leaf-litter"), the species that inherited subterranean foraging from the common ancestor of the genus *Dorylus *("original subterranean"), and the *Dorylus s.s*. species that adopted subterranean foraging secondarily ("reversal subterranean"). Four key morphological traits were analysed: Maximum head width (HWmax), mandible length (ML), antennal scape length (SL), and hind leg length (HLL). Niche category had a significant effect on all traits (Nested ANOVA, p < 0.025 for all traits). Significant differences (Tukey's Studentized Range Test, p < 0.05) are indicated by different letters.

The variation in maximum head width, antennal scape length, mandible length, and hind leg length was strongly correlated with foraging niche (Nested ANOVA, p < 0.025 for all traits). When comparing these traits between the species that reversed to subterranean foraging from leaf-litter foraging and the extant leaf-litter species, we found that all measurements were significantly smaller in the former category (Fig. 3; Tukey's Studentized Range Test, p < 0.001 in all cases). In fact, maximum head width, antennal scape length and hind leg length were all intermediate between the extant leaf-litter foragers and the originally subterranean species, while mandible length was even smaller in the reversed than in the originally subterranean species. With the reversal to subterranean foraging, species of the subgenus *Dorylus s.s*. thus secondarily evolved a similar morphology in four key traits related to foraging performance and these convergent adaptations shifted trait values fully back to or even beyond the values that characterize the species that inherited subterranean foraging directly from the common ancestor of all *Dorylus s.l*. species.

## Discussion and Conclusion

This study demonstrates that the currently recognized subgenera *Anomma *and *Dorylus s.s*. are not monophyletic. The driver ants of the subgenus *Anomma *clearly form a separate clade from the *Anomma *species that forage in the leaf-litter, which are more closely related to the subterranean species of the subgenus *Dorylus s.s*. (Fig. 1). The confusion about the subgenera *Anomma *and *Dorylus s.s*. can be traced back to the original descriptions of the leaf-litter *Anomma *species *D. kohli*, *D. gerstaeckeri*, and *D. emeryi *and of the subterranean *D. (Dorylus) spininodis*. Already Wasmann [11] pointed out that the workers of *D. kohli *seemed transitional between driver ants and *Dorylus s.s*. in both morphology and foraging habits. Because he assumed that the largest workers, which were not available to him for examination, would resemble those of driver ants, he assigned the species to *Anomma *[11]. Similarly, Emery [12] noted that workers of *D. gerstaeckeri *did not fit well with his diagnosis of *Anomma *and our present findings indicate that the male that he described as *D. (Dorylus) gribodoi *might in fact be the male of *D. (Anomma) gerstaeckeri *(Fig. 1, Table 1; although the genetic distance does not fall below our conservative cut-off value). Furthermore, Mayr [13] described *Dorylus emeryi *without reference to any subgenus, but the species was later assigned to *Anomma *without explicit justification [6,14]. Finally, males of *D. kohli *have been collected together with workers by J. van Boven and are now shown to be identical to males that have originally been described as a *Dorylus s.s*. species (W. Gotwald and CS, unpublished). It has thus always been acknowledged that males of leaf-litter *Anomma *species are morphologically more similar to males of the subgenus *Dorylus s.s*. than to the males of *Anomma *driver ants, but their correct associations were not established until the present study.

At the same time spurious assumptions and imprecise subgenus definitions have led to the incorrect assignment of the workers of leaf-litter species to the subgenus *Anomma*. While the workers of *Dorylus spininodis *(along with those of *D. politus*, which could not be included in this study) were recognized as clearly distinct from the *Dorylus s.s*. species of the "*D. affinis *species group" because they lack caudally projecting spines at the frontal carinae (a pair of longitudinal ridges between the antennal sockets and the anterior sclerite), the species was nevertheless assigned to the subgenus *Dorylus s.s*. [15]. We thus conclude that the subgenera *Anomma *and *Dorylus s.s*. are not monophyletic, that suspicions of this have existed for a long time but only make sense in light of our present data, and that the current subgenus classification cannot be upheld. We will formally propose the necessary taxonomic and nomenclature changes elsewhere.

Our results further show that the differences in life-style between *Dorylus s.l*. species are not merely the result of a simple unidirectional development from subterranean foraging to surface swarm raiding, as has been assumed so far [16], but that there has also been a reversal from leaf-litter foraging to subterranean foraging (Fig. 2). Previous analyses of *Dorylus s.l*. worker morphology already showed that shifts in foraging niche are correlated with changes in several morphological adaptations [17]. Longer hind legs increase running speed on the soil surface and allow the ants to carry larger prey items [18], whereas shorter legs are probably adaptive in subterranean species which hunt in narrow tunnels [19]. Similarly, larger heads can accommodate larger mandibular muscles so that workers can bite more fiercely [20]. Large heads and mandibles are thus likely to be adaptative in driver ants, which have to defend their nests and foraging or migration columns against vertebrate predators (such as chimpanzees, gorillas, Jackson's mongooses and pangolins) [1,21] and that benefit from being able to cut up larger prey. Longer antennae, on the other hand, may allow workers to follow pheromone trails at higher speed while avoiding collisions [22], which seems adaptive on open trails with a high volume of traffic. While an earlier analysis [17] treated all species with subterranean foraging as a single monophyletic group, our present phylogenetic study shows that the subterranean *Dorylus s.l*. species consist of two clades and that one of them has secondarily re-evolved subterranean foraging and many of the morphological adaptations to an entirely subterranean lifestyle (Fig. 2, Fig. 3). Our present findings thus give strong independent support for the inference of Schöning et al. [17] that many worker allometries of army ants represent adaptations to specific foraging niches, as we now know that the subterranean syndrome evolved twice convergently.

Our present study establishes that subterranean foraging is ancestral in *Dorylus s.l*. and that leaf-litter foraging and surface swarm raiding originated later. The imprecise age estimates resulting from the lack of *Dorylus s.l*. fossils make inferences regarding environmental conditions at the time of ecological niche shifts speculative. It is nevertheless interesting to note that the transition to surface swarm raiding and the reversal to subterranean foraging may have occurred at approximately the same time (Fig. 2). This raises the interesting question why one group of army ants reverted to the subterranean foraging niche, which was already occupied by distant relatives, roughly at the same time that a second group began to exploit the entirely new niche of surface swarm raiding. How foraging niches evolved in the other army ant lineages and whether the common ancestor of all army ants also hunted in the soil is as yet unknown. This information, combined with data on prey spectra and distributional ranges, will be crucial to infer under which conditions the army ant life-style evolved, which general factors promoted the subsequent niche shifts in different lineages, and where these transitions took place. Finally, detailed comparative analyses of worker morphology in the other army ant clades are needed to establish whether they also show correlated shifts in foraging niche and morphological traits of workers.

## Methods

### Taxon sampling

Our sample for DNA sequencing consisted of a total of 38 *Dorylus s.l*. specimens, both males and workers, representing all six recognized subgenera (see Table 2). *Eciton burchellii *(Ecitoninae) and three species of *Aenictus *(Aenictinae) were used as outgroups, but no fresh material of Aenictogitoninae was available. All specimens were collected and stored in 96% ethanol until DNA extraction.

### Molecular protocols

DNA was extracted from ant legs using the DNeasy^® ^kit from QIAGEN^®^. Two fragments from the mitochondrial *cytochrome oxidase I *(*COI*) gene and one fragment from the mitochondrial *cytochrome oxidase II *(*COII*) gene were amplified in standard polymerase chain reaction (PCR), using primers LCO/HCO [23] and Jerry [24]/Ben [25] for *COI *and primers tRNALeu/Barbara [24] for *COII*. In addition, we amplified a fragment of the nuclear *wingless *(*wg*) gene using primers wg1/wg2 [26]. The tRNALeu primer (5' CAGATTAGTGCAATGAATTTAAGT 3') was specifically designed for this study to replace primer George [24] to avoid the amplification of pseudogenes [27]. The annealing temperature was 45°C for all mitochondrial primers and 58°C for *wg*. We used a concentration of 2.5 mM MgCl_2 _in all reactions. PCR products were purified using a MicroSpin^® ^kit from Omega Bio-Tek. Automated fluorescent dye sequencing was conducted on an Applied Biosystems 3130 × l Genetic Analyzer or reactions were sent to a sequencing facility (MWG-Biotech) using the forward primer and, in many cases, also the reverse primer. In several cases where clean sequences could not be obtained due to co-amplification of probable pseudogenes, PCR products were cloned using the TOPO TA Cloning^® ^kit from Invitrogen^® ^and sequences were obtained from one or several clones. We generated a final concatenated alignment of 1957 base pairs (bp) of sequence data, consisting of 1007 bp from *COI*, 547 bp from *COII*, and 403 bp from *wg*. Of these, 923 sites were variable and 740 sites were parsimony informative. Sequences have been deposited in GenBank and accession numbers are given in Table 2. All sequences used in this study are new, except for *D. orientalis *and the *wg *sequences for *D. laevigatus*, *A. aratus*, and *A. laeviceps*, which are from Brady [2].

### Phylogenetic analyses

Several sequences with frame-shift mutations were discarded from the alignment as non-functional pseudogenes. The remaining DNA sequences were translated into amino acid sequences using the program MEGA 2.1 [28]. This did not reveal any stop codons that could have been indicative of additional pseudogenes, but made us exclude one more sequence from the alignment, because of an extremely high rate of unique amino acid substitutions (GenBank accession number [EF413886]). For several driver ant species, we obtained two alternative, potentially functional sequences for *COII*. One of them (obtained with primers George/Barbara) was identical across all species, indicating that it represented a nuclear pseudogene with a much lower mutation rate (GenBank accession number [EF413885]) [27], which we thus excluded. The second (obtained with primers tRNALeu/Barbara) was variable across species to an extent similar to what we observed for the other mitochondrial sequences and was thus assumed to be the true mitochondrial sequence.

Because all sequences are protein coding, alignment could be done unambiguously by eye in a text editor. To detect additional potential pseudogenes that would confound phylogenetic inference and to evaluate congruence among data sets, we conducted independent MP analyses of the single gene fragments with 1000 bootstrap replicates using heuristic searches, ACCTRAN character optimization and tree-bisection-reconnection branch swapping with steepest descent in PAUP*4.0b10 [29] with *E. burchellii *and three *Aenictus *species (Table 2) as outgroups. All well supported relationships were highly concordant across the different data partitions, except for two relationships within the *Anomma *driver ant clade (results not shown). This ambiguity was probably due to sequences for a number of driver ant taxa being unavailable in some of the data partitions as they had been excluded as probable pseudogenes (Table 2), which affected the resolution of relationships within that clade. However, some ambiguity in the precise relationships within the driver ants does not affect the overall conclusions of our study. We thus combined the data in a full evidence approach for all further analyses. The concatenated dataset was subjected to MP analysis in PAUP* as has been described for the single data partitions.

The best fitting model of DNA evolution for Bayesian analyses was selected for each codon position for both mitochondrial (*COI *+ *COII*) and nuclear (*wg*) sequences with MrModeltes v2 [30], comparing 24 nested models with hierarchical likelihood ratio tests (hLRT). The best model to explain the data was a general time reversible model with an estimated proportion of invariable sites and gamma distributed rate heterogeneity (GTR + I + G) for first and second mitochondrial codon positions, a GTR + G model for third mitochondrial codon positions, a HKY model for first nuclear codon positions and a K80 model for second and third nuclear codon positions. Bayesian analyses were performed with MrBayes v3.1.2 [31]. To assure convergence of Markov Chain Monte Carlo runs we repeated the analysis three times beginning with independent starting trees. In each analysis, one cold and three heated chains were run in parallel for 1 × 10^6 ^generations and trees were sampled every 100 generations. The first 1001 trees were discarded as burnin and the consensus tree was calculated from the remaining 9000 trees in PAUP*4.0b10 [29]. *E. burchellii *was used as the single outgroup in MrBayes v3.1.2 [31], following [2-4].

For ML analyses, the best model of DNA evolution for the concatenated dataset was chosen among 56 nested models with Modeltest 3.7 [32]. The best model to explain the data, both according to hLRT and the Akaike information criterion, was a general time reversible model with an estimated proportion of invariable sites and gamma distributed rate heterogeneity (GTR + I + G). ML searches were performed in GARLI v0.942 [33], which implements the GTR + I + G model as default, using the same outgroups as in MP analyses. The run was repeated three times from random starting trees using default termination conditions. GARLI v0.942 [33] was also used to generate 1000 ML nonparametric bootstrap replicates which were used to calculate a majority rule consensus tree in PAUP*4.0b10 [29].

### Male-worker associations

We computed uncorrected pairwise distances of mitochondrial DNA sequences between male and worker specimens in MEGA 2.1 [28] when the phylogenetic analyses indicated that they were closely related. We compared these distances to the genetic distance between two specimens of *D. (Anomma) molestus *collected from the same population at Mt. Kenya to identify a conservative cut-off point for inferring whether males and workers likely belonged to the same species.

### Reconstructing evolutionary transitions in foraging niche

For the reconstruction of evolutionary transitions in foraging niche we excluded all males from the dataset, because the foraging niche of their associated workers was unknown (they had been light trapped) and in most cases the species was likely to be represented also by a worker specimen for which we had that ecological information. Data on foraging niche were based on direct observations or on information from the literature [17]. We investigated the evolution of foraging niche in *Dorylus s.l*. army ants under MP using the computer program MacClade 4.0 [34] on the Bayesian topology obtained from MrBayes v3.1.2 [31]. The latter program was also used to obtain Bayesian posterior probabilities of foraging niche at different nodes. Foraging niche was added as a standard character to the data matrix in a separate partition while constraining one node of interest in each individual run. We used the same model and chain parameters that have been described above. Ordered character states for foraging niche (subterranean – leaf-litter – surface swarm raiding) were assumed in all analyses, because it is most plausible that army ants adapt only gradually to more exposed foraging strata.

### Morphological analyses

To evaluate the hypothesis that the relative dimensions of key morphological traits represent adaptations to different foraging niches, we examined the following traits in workers of thirteen species (Table 3): Maximum head width (the maximum measurable width across the head), mandible length (the distance between the apex of the left mandible and the proximal point of the ventral ridge when the mandibles are fully opened with forceps), antennal scape length (the maximum straight line scape length excluding the basal condyle), and hind leg length (the maximum length of the left hind leg from trochanter to tarsal tip in dorsal view with the leg fully extended).

**Table 3:** Samples used for morphometric measurements.

Species	Sub-genus	Foraging niche	Number of specimens	Collection locality
terrificus	An.	surface swarms	110	Kibale (Uganda)
molestus	An.	surface swarms	102	Mt. Kenya (Kenya)
mayri	An.	surface swarms	106	Bossou (Guinea)
arcens	An.	surface swarms	106	Taï (Ivory Coast)
emeryi	An.	leaf-litter	54	Taï (Ivory Coast)
gerstaeckeri	An.	leaf-litter	104	Taï (Ivory Coast)
kohli	An.	leaf-litter	102	Kibale (Uganda)
braunsi	Do.	subterranean	50	Kakamega (Kenya)
sp. 1	Do.	subterranean	102	Mt. Kenya (Kenya)
sp. 1	Rh.	subterranean	102	Mt. Kenya (Kenya)
sp. 2	Rh.	subterranean	102	Taï (Ivory Coast)
sp. 1	Ty.	subterranean	51	Gashaka (Nigeria)
cf. vishnui	Al.	subterranean	51	Poring (Malaysia)

Abbreviations are as in Table 2.

For these morphometric analyses we selected about 100 workers (preserved in 70% ethanol) from at least three colonies (50 workers from a single colony if insufficient samples were available) from the same study site for each species to cover the entire worker size range as much as possible. Ideally, workers from more colonies per species should have been included in our analysis to better cover the total intraspecific variation of morphological traits, but this was not possible because most species are only rarely collected so that larger samples are not available. We assume, however, that our morphological analysis based on a few colonies is fairly representative for the species as a whole, because measurement data on driver ants show that variation within and between populations of the same species is much less than variation between species (C. Schöning, unpublished). All measurements were taken using a MS 5 Leica stereomicroscope fitted with an ocular micrometer, using methods recommended by Seifert [35] to minimise measurement errors (magnifications 10× – 64×). Dry mass (measured after drying specimens at 60°C for 48 h) was used as an indicator of overall body size. The statistical analysis followed Schöning et al. [17]. In order to compare the relative size of traits between workers of different species we first established the best fit model for raw linear data from all species combined over their common body size range (0.29 mg – 1.50 mg) as a function of dry mass^1/3 ^by stepwise multiple regression. The relative residuals (absolute residuals divided by the predicted values) from this common regression model were then compared in a Nested ANOVA with foraging niche category (original subterranean – leaf-litter – surface swarm raiding – reversal subterranean) as a fixed factor and species as a random factor nested within foraging niche category. Tukey's Studentized Range (HSD) Test was employed to examine differences between categories. Statistical analyses of morphometric data were performed with SAS (Version 9.1).

### Divergence dating

The same dataset and topology that were used to reconstruct evolutionary transitions in foraging niche (see above) were also used for divergence dating analysis. Branch lengths were estimated in PAUP*4.0b10 [29] under maximum likelihood with a GTR + I + G model of DNA substitution. The model with a molecular clock enforced (-ln 15494.5) was compared with a model without a molecular clock (-ln 15433.9) using a likelihood ratio test. The null model of rate constancy (the molecular clock) was rejected at p < 0.001 (28 df). Prior to dating analyses, *E. burchellii *was pruned from the phylogram to avoid ambiguity about the placement of the root. Dating analyses were conducted with the program r8s v. 1.7 [36], using penalized likelihood [37], the TN algorithm and an additive penalty function. The smoothing parameter was chosen using cross-validation of parameter values as implemented in r8s v. 1.7 [36]. Solutions were checked with the "checkGradient" command. Because no *Dorylus s.l*. fossils are known that could have been used to calibrate the analysis, we had to rely on earlier published dates that were calibrated with fossils in the frame of a larger scale molecular phylogeny of army ants and their relatives [2]. To cover a range of reasonable dates for each node, we ran three analyses, using the mean, upper and lower bound of the 95% confidence interval for the most recent common ancestor of *Dorylus s.l*. (45.6, 64.0, 30.0 million years ago, respectively) [[2], Brady pers. comm.] to fix the respective node.

## Authors' contributions

DJCK carried out the DNA sequencing work and the analyses of the molecular data, and drafted a first version of the manuscript. CS collected most samples, measured the morphological traits and analysed them. LBV participated in the analysis of morphological data. JJB coordinated the study and helped to design the format of the manuscript. All authors participated in designing and planning the study, in writing the various versions of the manuscript, and they all read and approved the final version.
